# From identity to function: Unveiling the cellular complexity of hypothalamic feeding circuits

**DOI:** 10.1007/s11154-025-10004-7

**Published:** 2025-10-28

**Authors:** Sheridan H. Littleton, John A. Tadross, Giles S. H. Yeo

**Affiliations:** 1 Medical Research Council Metabolic Disease Unit, Institute of Metabolic Science-Metabolic Research Laboratories, University of Cambridge, Cambridge, UK; 2 Department of Histopathology and Cambridge Genomics Laboratory, Cambridge University Hospitals NHS Foundation Trust, Cambridge, UK

**Keywords:** Hypothalamus, Feeding behavior, Transcriptomics, Single-cell, Obesity, Atlas

## Abstract

The hypothalamus is arguably the most complex part of the brain, with the greatest heterogeneity of cellular populations. It is an integration center for peripheral signals and external stimuli and plays a key role in coordinating a myriad of fundamental biological functions, including energy homeostasis. To fully understand how the hypothalamus functions, we first need to unravel cellular heterogeneity of this brain region. In hypothalamic research, the vast majority of ‘ground truths’ have, until recently, emerged from low-throughput murine studies. However, the advent of high-throughput, single-cell approaches has dramatically altered the landscape and allow characterization of gene and protein expression, epigenomic features, cell morphology, and spatial organization. Recently, three-dimensional transcriptomic atlases of the macaque and human hypothalamus have been published and serve as growing resources for understanding hypothalamic cell types and organization in both healthy and disease states. Hypothalamic function is still primarily studied with the use of model systems, where cell culture methods are especially suitable for investigating molecular mechanisms, while animal models provide the opportunity to disentangle complex neural circuits as well as measure behavior and physiological changes. Here, we review what the latest technological advancements and functional discoveries have revealed to us about the hypothalamus, with a focus on feeding behavior.

## Abbreviations

RNA-seq: RNA sequencing
bacTRAP: Bacterial artificial chromosome-translating ribosome affinity purification
AVP: Arginine vasopressin
AgRP: Agouti-related peptide
NPY: Neuropeptide Y
MERFISH: Multiplexed error-robust fluorescence *in situ* hybridization
TRISCO: Tris buffer-mediated retention of *in situ* hybridization chain reaction signal in cleared organs
DBiT-seq: Deterministic barcoding in tissue sequencing
LHA: Lateral hypothalamic area
VMH: Ventromedial hypothalamic nucleus
POMC: Pro-opiomelanocortin
PVN: Paraventricular nucleus
SUM-PAINT: Secondary label-based unlimited multiplexed points accumulation for imaging in nanoscale topography
cycleHCR: Cycle hybridization chain reaction
INF: Infundibular nucleus
ARC: Arcuate nucleus
CRH: Corticotropin-releasing hormone
BMI: Body mass index
DMH: Dorsomedial hypothalamic nucleus
GLP-1: Glucagon-like peptide-1
GIP: Gastric inhibitory polypeptide
shRNA: Short hairpin RNA
m^6^A: N^6^-methyladenosine
RAMPANT: Rabies afferent mapping by poly-A nuclear transcriptomics
cAMP: Cyclic adenosine monophosphate
MC4R: Melanocortin-4 receptor
α-MSH: Alpha-melanocyte stimulating hormone
PVN^MC4R^: MC4R neurons in the PVN
BDNF: Brain-derived neurotrophic factor
VMH^BDNF^: BDNF neurons in the VMH
Gα_q_: Guanine nucleotide-binding protein G alpha q
Gα_s_: Guanine nucleotide-binding protein G alpha s
ERα: Estrogen receptor α

## 1 Introduction

Diet-related illnesses are arguably the greatest threat to public health of the 21st century, with the prevalence of obesity increasing dramatically over the past three decades [1, 2]. Understanding the mechanisms controlling feeding behavior will be important for informing any strategy to improve our health in this current food environment. The hypothalamus is a small, yet very complex, part of the brain with diverse functions, including regulation of feeding, energy expenditure, thirst, body temperature, sleep, circadian rhythms, puberty, and mood [3]. This review will focus on hypothalamic regulation of appetitive and metabolic traits. Genetic and molecular studies have uncovered key cells within the hypothalamus that integrate external stimuli and nutritional signals to regulate food intake and body weight. However, the inaccessibility of the human brain has, to date, meant our understanding of the hypothalamus has emerged primarily from murine studies. Investigating the complexity of the human hypothalamus at a single-cell level will enable better understanding of disease etiology, how current therapeutics are working, and possibly help discover new drug targets.

**Fig. 1:**
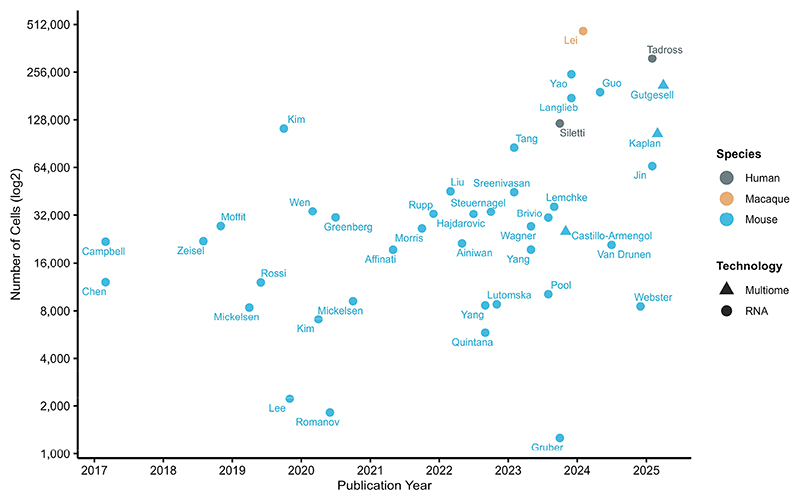
Exponential increase in hypothalamic single-cell sequencing datasets over time, where each point represents a published single-cell study, labelled by first author surname, and plotted by publication year and approximate number of hypothalamic cells profiled (log2). Color indicates species (human, macaque, or mouse) and shape denotes sequencing technology (multiome or RNA) [5, 15, 17, 18, 24–59]

Understanding the hypothalamus requires a precise characterization of its form, that is, its diverse cell types and their organization. In this region of the brain, structure and function are closely entwined and accurate mapping of hypothalamic cell types is not merely descriptive—it is foundational. While many single-cell datasets have been generated across whole brains of various organisms [4–14], especially by groups such as the Allen Institute for Brain Science [15–18], BRAIN Initiative Cell Census Network [19, 20], Human BioMolecular Atlas Program [21], and Human Cell Atlas [22, 23], we see the need to focus specifically on the hypothalamus to increase the depth of data in such a heterogeneous brain region. A variety of high-resolution, single-cell techniques are now available to characterize cellular organization, gene and protein expression, neuron projections, and epigenetic features. As a result, we’ve seen an exponential increase in hypothalamic single-cell sequencing data in the past decade (Fig. 1). Here we describe how these methods have been or could be used to make substantial progress towards understanding hypothalamic cell types in mice, macaques, and humans, including the generation of three-dimensional atlases. We also discuss recent landmark functional discoveries in hypothalamic control of feeding.

## 2 Methods to characterize hypothalamic cell types

### 2.1 Cell mapping

#### 2.1.1 Whole-brain imaging

**Fig. 2:**
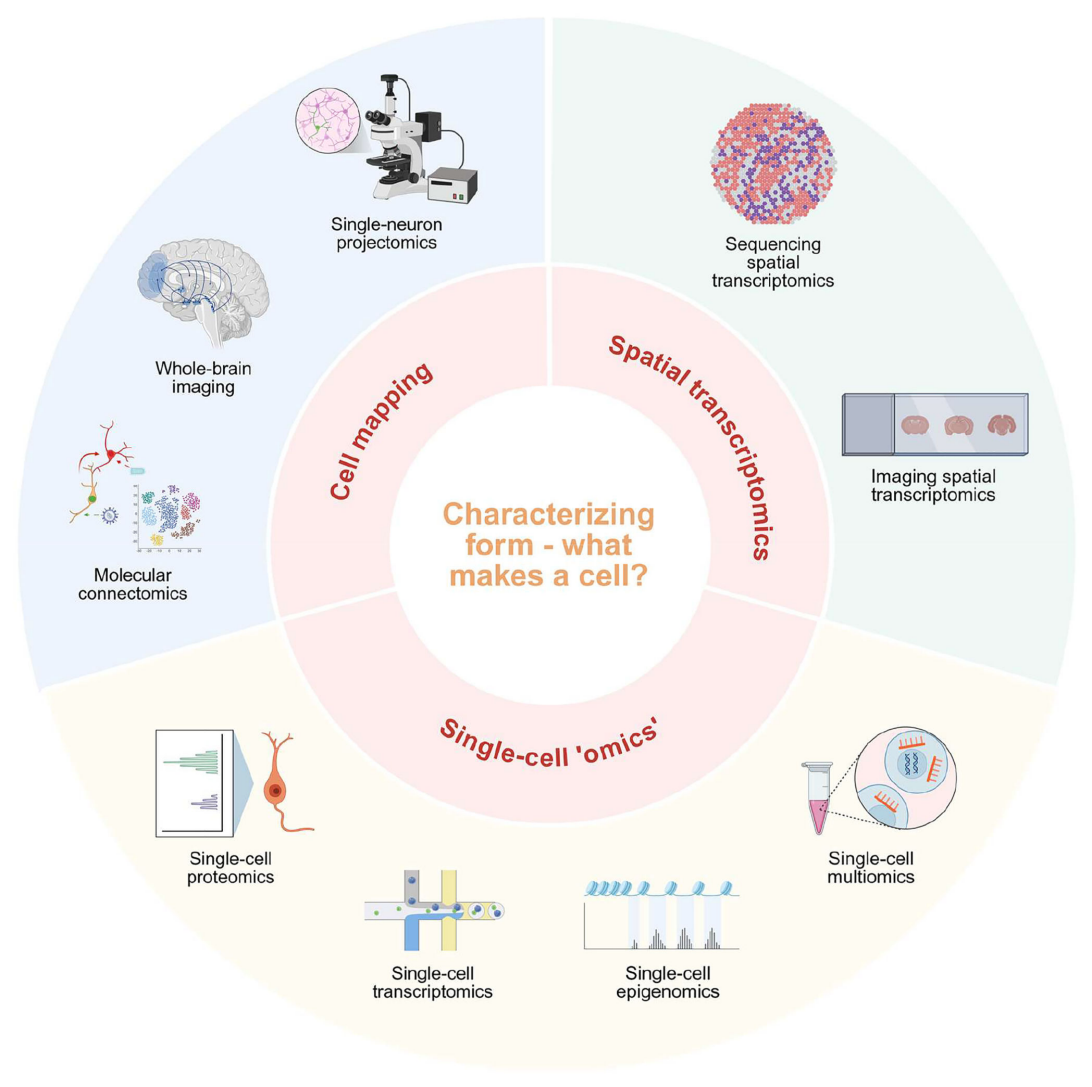
High-throughput technologies for defining cellular form, grouped into three methodological domains: cell mapping, single-cell ‘omics,’ and spatial transcriptomics

While the focus of this review is the hypothalamus, we must briefly discuss the utility of tissue clearing, immunolabeling, and whole-brain imaging to visualize molecular markers in a large, complex, three-dimensional space (Fig. 2). Tissues can be made transparent through a variety of methods, but this generally involves tissue fixation, permeabilization, decolorization, and refractive index matching to minimize light scatter [60–75]. Next, cell types of interest can be visualized either via genetically-encoded fluorescent reporters or fluorescently-labelled antibodies and then imaged within the context of the whole brain using modern, large-scale microscopy techniques, such as ultramicroscopy and light-sheet microscopy [61–75]. These approaches have allowed us to map cell-type distributions, cell morphology, neuronal connectivity, and neuronal activity in response to stimuli [61–63, 69, 71–75]. These techniques are more challenging in large, post-mortem, human brain tissues, but future studies may enable molecular reconstruction of the entire human brain [64, 65, 69, 70, 73].

#### 2.1.2 Single-neuron projectomics

It is crucial to understand complex axonal projection patterns to reveal brain regions that are targets of hypothalamic neuropeptides (Fig. 2). Genetically modified male mice and fluorescence micro-optical sectioning tomography have been used to map whole-brain projections of 7,180 individual hypothalamic peptidergic neurons [76]. These neurons expressed neuropeptide genes, namely *Agrp, Avp, Crh, Orexin, Oxt, Pmch, Pomc, Vip, Adcyap1, Nts, Pdyn, Penk, Sst, Tac1, Tac2*, and *Trh*, relevant to hypothalamic function [76]. The axon projection patterns of these neurons were classified into 31 distinct types, where types 1-15 targeted areas such as the hypothalamus, cortex, pallidum, thalamus, and striatum and types 16-31 targeted areas in the midbrain, pons, and medulla [76]. Most projectome types exhibited regionally biased soma distributions in one or more hypothalamic nuclei and specific neuropeptide expression patterns [76]. For example, midbrain-projecting neurons were enriched in the lateral hypothalamic area (LHA) and ventromedial hypothalamic nucleus (VMH), and those neurons with somas in the VMH that had projections to the midbrain were enriched with *Pdyn, Adcyap1*, and *Tac2* expression [76]. Focusing on neurons involved in appetite regulation, pro-opiomelanocortin (POMC) neurons had significant projections to the midbrain, pons, and medulla while AgRP neurons projected within the hypothalamus to regions including the paraventricular nucleus (PVN**)**, anteroventral periventricular nucleus, and median eminence [76]. AgRP neurons, as well as many other types of hypothalamic peptidergic neurons, projected to multiple targets, showing that a single axon may be able to simultaneously influence multiple brain areas [76]. Another group used the same methods, focusing only on oxytocin neurons in the PVN and similarly found distinct types of projections, regional targets, and gene expression patterns [77]. Taken together, this work illustrates the complexity of hypothalamic axonal projections, both locally within the hypothalamus and long range [76, 77]. Future studies will also determine how morphological subtypes correlate to or further refine cell populations identified by single-cell and spatial transcriptomics.

#### 2.1.3 Molecular connectomics

Sequencing-based methods can also be used to identify synaptic connections between neurons [59] (Fig. 2). A method upstream of single-nucleus RNA sequencing (RNA-seq), rabies afferent mapping by poly-A nuclear transcriptomics (RAMPANT), is used to identify connections with specific neurons of interest and has been applied to AgRP neurons in the mouse hypothalamus [59]. This study involved genetically-modified mice that expressed a virus receptor in AgRP neurons, which were then also injected with viruses that can spread trans-synaptically and were modified to express a nuclear-localized mCherry fluorescent protein [59]. This resulted in fluorescently-labeled mouse neurons either expressing *Agrp* or synapsing on AgRP neurons, whose nuclei were then captured with fluorescence-activated sorting before single-nucleus RNA-seq [5, 59]. This selective sequencing enabled the identification of 14 types of neurons that synapse on AgRP neurons, including a previously uncharacterized inhibitory *Trh*^+^/*Glp1r*^+^/*Lepr*^+^ neuron population in the caudal ARC that provides direct input to AgRP neurons, decreasing feeding [59]. Crucially, these neurons also directly responded to liraglutide and contributed to its satiation and weight loss effects [5, 59]. In the future, RAMPANT can be applied to target other types of neurons to identify novel regulatory partners and mechanisms.

### 2.2 Single-cell ‘omics’

#### 2.2.1 Single-cell transcriptomics

Single-cell and single-nucleus RNA sequencing methods are used to study the transcriptome of individual cells, which is crucial to understand patterns of gene expression in a heterogeneous and sexually dimorphic tissue, such as the hypothalamus (Fig. 2). To obtain cells or nuclei, tissue is first dissected either macroscopically or microscopically, which can be aided by sophisticated methods like laser capture microdissection [78] or expression microdissection [79] to further select specific cell types from the tissue [80]. Next, the tissue is dissociated into a single-cell or single-nucleus suspension with enzymes, detergents or mechanical force, which is challenging and can introduce artifacts, such as stress-induced *ex vivo* changes in gene expression [81] or bias towards capturing certain cell types that would skew our understanding of cellular composition [82]. Single-nucleus methods are more commonly used with brain tissue and have advantages of being compatible with archived frozen samples and decreasing dissociation bias and transcriptional stress responses, while maintaining comparable gene detection ability compared to single-cell methods [83, 84]. When working with human tissues, pre- and post-mortem tissue handling can also introduce artifacts [81], which are difficult to prevent but should be considered during experiment design and interpretation of results.

We previously published HypoMap, a single-cell transcriptomic atlas of the mouse hypothalamus [53]. This atlas is composed of 384,925 cells from 18 independent single-cell or single-nucleus RNA-seq datasets [24, 44–52, 54–57, 85, 86] across various hypothalamic regions, from the preoptic area to the ventroposterior hypothalamus [53]. We broadly annotated cell populations as excitatory glutamatergic neurons, inhibitory GABAergic neurons, astrocytes, ependymal cells, oligodendrocytes, microglia, and endothelial cells [53]. At higher resolution, we identified 465 distinct cell clusters, highlighting the diversity of cell types in the hypothalamus [53]. Focusing on neural populations involved in appetite and feeding behavior, utilizing a cell type-specific enrichment technique named bacterial artificial chromosome-translating ribosome affinity purification (bacTRAP) [87, 88], we identified *Glp1r*^+^ and *Pnoc*^+^ neurons and found that they are heterogeneous, both excitatory and inhibitory, and distributed across several hypothalamic regions [53]. Overall, HypoMap serves as a dynamic resource to help unravel the complexity of the hypothalamus and identify druggable targets for metabolic disorders.

#### 2.2.2 Single-cell proteomics

Measuring protein expression is especially valuable for identifying functional differences between cell types as RNA expression does not always strongly correlate with protein expression [89, 90] (Fig. 2). Mass cytometry has been used to build a single-cell protein atlas of the developing mouse brain [91]. Secondary label-based unlimited multiplexed points accumulation for imaging in nanoscale topography (SUM-PAINT) is a high-throughput imaging-based method that has been used to atlas proteins in mouse neurons at single-protein resolution [92]. Cycle hybridization chain reaction (cycleHCR) is a new imaging-based technique that can be used to characterize both RNA and protein expression in the same tissue specimen and has been applied to mouse hippocampus [93]. Single-cell proteomics has not yet been applied to the hypothalamus but would be a valuable endeavor to provide insight into hypothalamic complexity.

#### 2.2.3 Single-cell epigenomics

Other single-cell and spatial methods can be used to characterize epigenomic features, such as chromatin accessibility and architecture, DNA methylation, and histone modifications, providing a more complete picture of the diversity and sexual dimorphism of hypothalamic cell types [94–101] (Fig. 2). Epigenomic methods have also been combined with others to create multiomic datasets to integrate single-cell DNA methylation patterns and neuronal projections in the mouse brain [9], identify sex-specific methylation patterns during mouse arcuate nucleus development [102], or characterize cell type- and sex-specific regulatory elements in the human hypothalamus [103] (Fig. 2). Similar to proteomics, there is a scarcity of hypothalamic single-cell and spatial epigenomic data to date, but it would be a welcome addition to ongoing atlasing efforts.

### 2.3 Spatial transcriptomics

While single-cell transcriptomic techniques have the advantage of easily characterizing hundreds of thousands of cells at once, they start with dissociating the tissue which eliminates all spatial context. The advent of spatial transcriptomics techniques has allowed researchers to measure gene expression within the tissue structure, providing extra insight into the complexity and organization of the hypothalamus and other tissues. Early spatial transcriptomic techniques had resolutions often larger than a single cell, so data points reflected gene expression of multiple cells. As technology improves, we are moving from resolution that captures several cells at a time to sub-cellular resolution.

High-throughput spatial transcriptomics methods are either imaging- or sequencing-based (Fig. 2). Xenium [104, 105], multiplexed error-robust fluorescence *in situ* hybridization (MERFISH) [15, 48, 106–108], and Tris buffer-mediated retention of *in situ* hybridization chain reaction signal in cleared organs (TRISCO) [4] involve the use of fluorescently-labeled probes and microscopy to measure gene expression within tissue. Other methods that use sequencing of either RNA probes, RNAs, or spatial barcodes include Visium [25, 105, 109–111], Slide-seq [5, 109, 112, 113], Stereo-seq [58, 109, 114–116], Slide-tags [95, 109], and deterministic barcoding in tissue sequencing (DBiT-seq) [109, 117]. Spatial transcriptomics methods have been used with mouse [48, 107, 114], rat [110, 111], macaque [58], and human [25, 105] hypothalamus, other brain regions or whole brain [4, 5, 15, 95, 106–109, 112, 113, 116, 117], and embryos [109, 115, 117]. Spatial transcriptomic data can be used to answer questions about development [113–115], puberty [110], aging [108], brain injury [112], disease (e.g. epilepsy [104], tumors [95, 117]), and sex-differences [105, 111], or to build cellular atlases [4, 5, 15, 25, 48, 58, 116].

## 3 Building a three-dimensional multiomic hypothalamus atlas

### 3.1 Non-human primate hypothalamus

A single-cell spatial atlas of the hypothalamus from non-human primates was recently published [58]. Non-human primates are a strong candidate model system as they are likely more clinically relevant for humans than mice. This atlas includes transcriptomic data (single-nucleus RNA-seq and spatial Stereo-seq) from eight adult male macaques (*Macaca fascicularis*) and is comprised of three controls, three with obesity due to high-fat diet, and two with non-obese, sporadic type 2 diabetes [58]. The atlas contains 465,632 hypothalamic cells, which were categorized as neurons (glutamatergic, GABAergic, histaminergic, and oxytocinergic) or non-neuronal cell types (astrocytes, vascular cells, ependymal cells, tanycytes, microglia, oligodendrocytes, and oligodendrocyte precursor cells) [58]. The neurons displayed much heterogeneity, subclustering into 48 distinct groups [58]. Single-cell resolution spatial transcriptomic data revealed that many cell types display unique distributions across different regions [58]. Some results were not surprising, for example, observing ependymal cells primarily lining the edge of the third ventricle or an enrichment of oligodendrocytes insulating axons in the optic tract [58]. Most of the neuronal subclusters were localized in distinct locations; *POMC*^+^/*TBX3*^+^ neurons were in the infundibular nucleus (INF) (similar to arcuate nucleus (ARC) in mice and humans) and *CRH*^+^/*RNPC3*^+^ and *OXT*^+^/*PTGFR*^+^ neurons in the PVN [58].

This atlas not only provides insights into the organization and function of distinct hypothalamic nuclei, but also allows for cross-species comparisons. Many neuronal populations were conserved between macaques and mice, such as arcuate AgRP neurons and paraventricular AVP neurons, but others were unique to either species, including corticotropin-releasing hormone (CRH) GABAergic neurons in mice compared to CRH glutamatergic neurons in macaques [58]. Another example of a species-specific gene expression pattern is *NTS* expression in ependymal cells in macaques and humans but neural expression in mice [58]. Characterizing differences between species will advance our understanding of evolutionary biology and inform how relevant non-human hypothalamic model systems are for functional experiments and drug testing.

Another strength of this non-human primate atlas is that it included macaques with obesity and type 2 diabetes and therefore allowed for investigation of cell type composition and transcriptional changes in response to metabolic disease [58]. The authors found the proportions of neuronal subtypes were largely unaffected by disease, whereas proportions of non-neuronal cell types showed modest shifts in diabetes and obesity. [58]. They also report that obesity and diabetes induced significant changes in gene expression, with diabetes influencing more unique genes than obesity. Obesity-specific transcriptional changes were largely in neuron populations expressing *AGRP, GHRH, POMC, GALP*, and *KISS1* in the INF [58]. Obesity and diabetes also corresponded to changes in gene expression in non-neuronal cell types, especially microglia and tanycytes [58]. Obesity decreased expression of genes involved in neurite growth and neural projections in the PVN and decreased neural signaling transmission cascade in the INF [58]. Transcriptional changes in response to diabetes were localized to both the INF and PVN, including in neuron populations expressing *AVP, NPY, CRH*, and *OXT* [58]. Diabetes increased expression of genes involved in immune response in the INF and decreased expression of genes in pathways related to adenosine triphosphate metabolic process [58]. These nuclei-specific changes highlight the need to study the functional complexity of the hypothalamus in the context of metabolic disease.

### 3.2 Human hypothalamus

We recently published a single-cell spatial atlas of the human hypothalamus, including 433,369 cells from single-nucleus RNA-seq of tissue from 11 donors with normal body mass index (BMI) and spatial transcriptomic profiling using 10X Genomics Visium of nine tissue sections spanning the antero-posterior extent of the hypothalamus [25]. These cells were broadly characterized as neurons, oligodendrocytes, astro-ependymal cells, and other non-neuronal cell types, such as microglia and endothelial cells [25]. These cells were then further characterized via multi-level clustering into five levels and 452 unique clusters to provide optimal granularity for further comparisons within such a complex tissue region [25]. The spatial and single-cell transcriptomic data was integrated to spatially map single-cell RNA-seq clusters to tissue sections throughout the hypothalamus, generating a three-dimensional atlas [25].

**Fig. 3:**
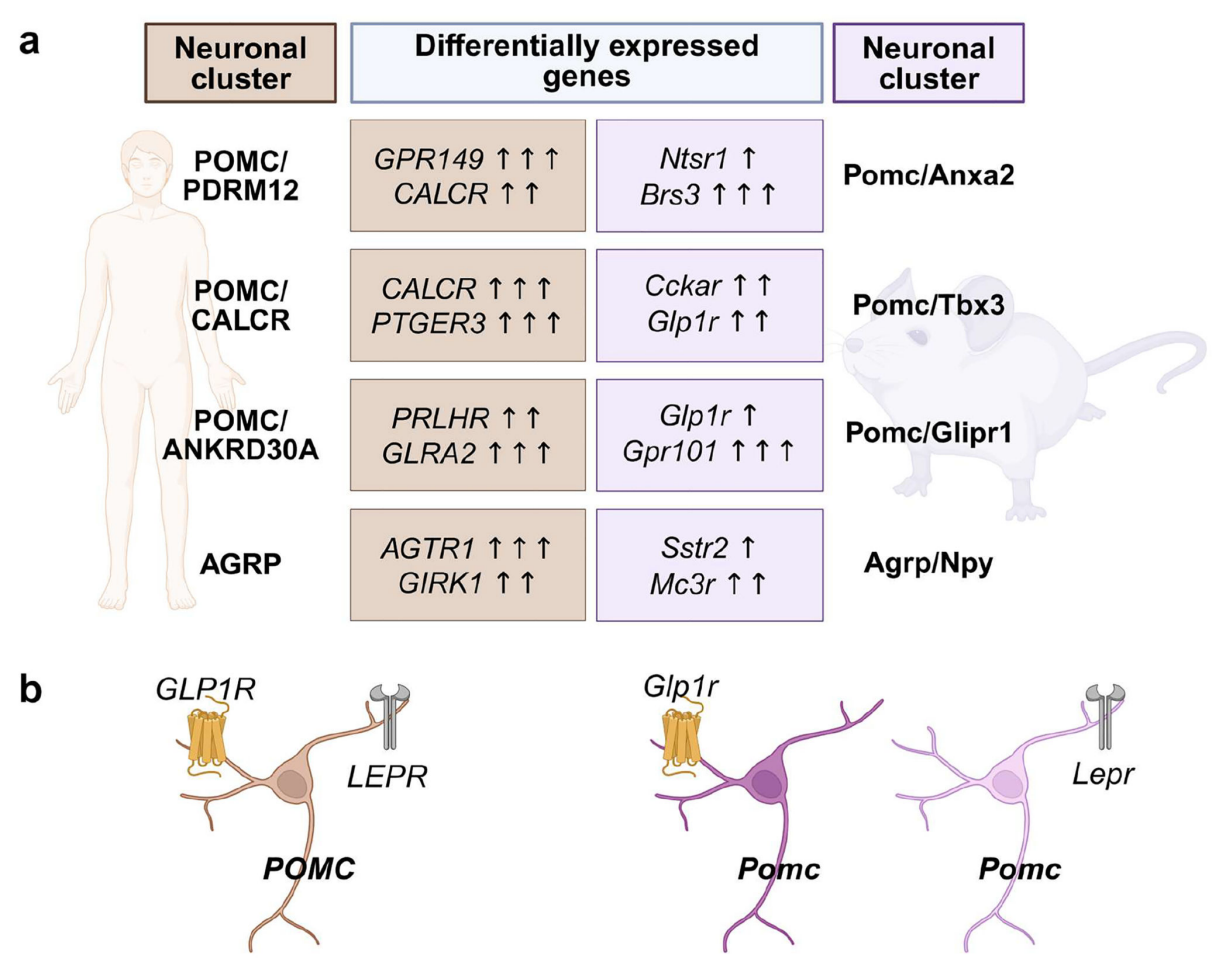
Examples of therapeutically pertinent differences in G-protein coupled receptor gene expression between human and mouse POMC and AgRP neurons. All gene names are italicized and human genes are all uppercase while mouse genes are in sentence case, in accordance with gene nomenclature guidelines for each species [120, 121]. Cell cluster names follow capitalization guidelines for each species but are not italicized to distinguish them from gene names. (a) Selected examples of receptor gene expression differences between matched human and mouse hypothalamic neuronal clusters, with cluster names simplified from the original publications [25, 53]. Where one human neuronal cluster corresponds to multiple mouse clusters (or vice versa), data are aggregated and cluster names reflect the dominant marker genes in the aggregated clusters (Online Resource 1). Arrows indicate fold difference in transcript expression levels: one arrow, ≥2-fold; two arrows, ≥10-fold; three arrows, ≥50-fold. (b) In the human hypothalamus, POMC neurons co-express *LEPR* and *GLP1R*, whereas in mice, *Lepr* and *Glp1r* mark transcriptionally and functionally distinct POMC subpopulations [25, 53, 119]

We compared our human and mouse single-cell hypothalamic atlases [25, 53] and identified clusters that had gene expression patterns that were unique to either species [25]. When considering neurons well-represented in both datasets, found in the ARC, VMH, and dorsomedial hypothalamic nucleus (DMH), 33% of clusters were human specific, highlighting species-specific differences in hypothalamic organization and function [25]. There were also differences in heterogeneity, for example, the DMH had greater cell type complexity in humans than in mice [25]. We investigated the conservation of G-protein-coupled receptors involved in the regulation of food intake and energy balance between mice and humans [25] (Fig. 3a, Online Resource 1). Notably, expression of leptin and NPY receptors in human *POMC*^+^/*PRDM12*^+^ neurons were conserved in mice, while cholecystokinin and bombesin receptors are expressed in mice but not in humans [25] (Fig. 3a). We also identified human cell clusters that expressed *GLP1R* and *GIPR*, receptors for glucagon-like peptide-1 (GLP-1) and gastric inhibitory polypeptide (GIP) incretin hormones and type 2 diabetes and obesity therapeutics [25, 118]. In humans, *GLP1R*^+^/*GIPR*^+^ cells mapped to the PVN and supraoptic nucleus and *GLP1R*^+^/*SIM1*^+^/*AVP*^+^ cells mapped to the PVN [25]. Several key cross-species differences were also observed; mouse *Sst*^+^/*Glp1r*^+^ cells closely correspond to a cluster in humans, but the human population does not express *GLP1R* and *SST*^+^/*GAL*^+^/*CALCR*^+^/*GLP1R*^+^ human cells closely match a cluster in mice, but the mouse population doesn’t express *Glp1r* [25]. Additionally, human POMC neurons co-express *LEPR* and *GLP1R*, whereas in mice, *Glp1r* and *Lepr* are expressed by transcriptionally and functionally distinct POMC neuron populations [25, 53, 119] (Fig. 3b). These findings underscore the importance of analyzing human-specific hypothalamic data, as key differences between species may limit the translational relevance of rodent models for understanding human metabolic disorders.

## 4 Methods to characterize hypothalamic function

Functional experiments are incredibly valuable to characterize the roles of specific brain regions, cell types, neural circuits, neuropeptides, genes, and mutations in influencing feeding behavior and obesity. These experiments require tractable cell culture or animal models where perturbations can be made. We describe recently published studies that have significantly contributed to our knowledge of hypothalamic function and highlight the utility of model systems. As more hypothalamic data is generated in several species and cross-species comparisons are made, we will better understand the translational potential of different models for understanding human health.

### 4.1 Cell culture models

Cell culture models are especially tractable tools to study hypothalamic function. Many hypothalamic immortalized cell lines have been established over the last 35 years and have been used to study a variety of functions [122]. More recently, protocols for stem-cell derived hypothalamic-like cells [123–130] and organoids [131–133] have been developed. Some advantages of hypothalamic cell culture models are that they are in greater supply than tissue samples and animals, have fewer restrictions on their use, and are more homogeneous and can therefore be used to study molecular mechanisms in one specific cell type. While cell culture models have their limitations, they are a valuable resource to help us understand complex brain regions and can complement experiments performed with other models.

#### 4.1.1 Genetic perturbations

Cell culture models present the opportunity to perform genetic perturbations in human hypothalamic cells that could not otherwise be done *in vivo*. These perturbations can be targeted to a gene [132, 134, 135] or a single variant [134, 136, 137]. A recent example of gene-level perturbation is a study that used short hairpin RNAs (shRNAs) to knockdown genes involved in writing and reading N^6^-methyladenosine (m^6^A) RNA modifications, *METTL14* and *YTHDC1*, in stem cell-derived ARC-like organoids. They found reduced proportions of POMC^+^ and OTP^+^ neurons and expression of POMC and NPY proteins in response to *METTL14* and *YTHDC1* knockdown, suggesting that m^6^A epitranscriptomic regulation plays a role in human arcuate neuronal development [132].

Exome- and genome-wide association studies have identified hundreds of coding and non-coding variants associated with BMI in human cohorts [136, 138–144]. These associations require functional validation and cell culture models can be particularly useful as effects of a single variant may be subtle, cell type-specific, or difficult to measure in whole animals [122, 145]. We recently explored the functional consequences of a BMI-associated protein-truncating variant in *BSN* using CRISPR-Cas9 editing and stem cell-derived hypothalamic-like neurons and identified changes in expression of genes involved in neurodevelopment, neurogenesis, and neuronal oxidative phosphorylation [136]. We also recently used the same methods to investigate the function of a BMI-associated non-coding variant in the 3’ untranslated region of *FAIM2* and found that the variant significantly decreased the proportion of neurons produced by the differentiation and caused cell type-specific changes in gene expression [137]. These studies highlight the utility of cell culture models to understand the molecular mechanisms of rare or common genetic variants that contribute to obesity risk.

#### 4.1.2 Patient-specific disease modeling

Induced pluripotent stem cells from patients with rare genetic obesity disorders have been differentiated to hypothalamic-like cells [146, 147] and organoids [131] to study the molecular basis of hyperphagic obesity in humans. Mutations in *BBS1* and *BBS10*, which cause Bardet-Biedl syndrome, did not affect neuronal differentiation efficiency but impaired neurite outgrowth, insulin signaling, and leptin signaling, decreased *POMC* expression and neuropeptide production, and caused longer primary cilia [147]. Mutations at the 15q11.2-q13 chromosome region, which cause Prader-Willi syndrome, did not affect stem cell arcuate neuronal differentiation efficiency [146]. However, Prader-Willi mutations caused ARC-like organoids to have neural proliferation and differentiation deficits along with other functional consequences, such as decreased frequency of neuronal firing, and defective leptin signaling and melanin-concentrating hormone release [131]. Such studies have also implicated genes, proteins and pathways that could contribute to obesity pathogenesis and warrant further functional investigation in cell or animal models [131, 146, 147].

### 4.2 Animal models

Animal models allow researchers to study the function of cells *in vivo* and in relation to their environment. Here we describe key studies that have used animal models to characterize hypothalamic regulation of appetitive and metabolic traits, with an emphasis on research published in the last two years.

#### 4.2.1 Diet-induced changes

The diet of animals can be manipulated to test the effects of nutrient content on hypothalamic cells. A recent study used single-nucleus RNA-seq to quantify changes in cell types and gene expression in the hypothalamus of young (postnatal day 15) male and female mice in response to maternal high fat diet during lactation [148]. 38,594 cells were identified as either neurons, astrocytes, tanycytes, ependymal cells, oligodendrocyte precursors, oligodendrocytes, microglia, stromal cells, or immune cells, with no significant difference in cellular composition between groups [148]. There was high heterogeneity in the neuron population which could be separated into 30 distinct subpopulations, again highlighting the complexity of the hypothalamus, even at postnatal day 15 [148]. Maternal high fat diet had the largest impact on neuronal gene expression and several subpopulations of neurons displayed different abundances, mostly in males, including arginine vasopressin (AVP)/retinoic acid receptor-related orphan receptor β, histaminergic, and agouti-related peptide (AgRP)/neuropeptide Y (NPY) neurons [148]. This study demonstrates the utility of single-cell RNA-seq and animal models to answer questions about hypothalamic function in response to nutrient intake.

#### 4.2.2 Sexual dimorphism

Regions of the hypothalamus, such as the VMH and ARC, are sexually dimorphic and contribute to sex-specific differences in the regulation of energy homeostasis [100, 101, 149–165]. Sex differences in metabolism and feeding behavior are largely modulated by sex hormones and their receptors [157], for example, estrogen receptor α (ERα) [156]. ERα signaling in the VMH regulates physical activity and thermogenesis but the neuron populations involved in this regulation have not been fully characterized [156]. Towards this end, recent work identified distinct neuron populations (marked by *Hpcal1, Tac1, Pdyn, Gal, Sst*, or *Rprm* expression) and sex-biased gene expression in the mouse VMH [156]. The *Sst* neuron population showed the largest overall transcriptional difference between males and females [156]. Fluorescence *in situ* hybridization demonstrated that *Tac1* and *Rprm* expression was enriched in females and *Pdyn* expression was enriched in males, but these sex differences were restricted to the ventrolateral region of the VMH [156]. The female-biased expression of *Tac1* and *Rprm* is established by ERα signaling during development [156]. However, male-biased expression of *Pdyn* is maintained by circulating testicular hormone during adulthood [156]. Reduction of *Rprm* expression increased core temperature in females and increased *Rprm* expression in males reduced core temperature [156]. Overall, this study advanced our understanding of how sexually dimorphic expression patterns are established and function to regulate energy expenditure [156].

#### 4.2.3 Neuron activity

Animal models provide the opportunity to measure neuron activity in response to complex sensory and metabolic cues. A recent study used long-term, continuous *in vivo* fiber photometry to record arcuate AgRP neuron activity in mice [166]. They found that AgRP neuron activity decreases before *ad libitum* feeding and in response to high-fat diet, increases rapidly due to fasting, and displays a circadian rhythm independent of feeding events [166]. They investigated the mechanism behind this daily neuronal activity pattern and determined that it is maintained by a neural circuit that links neurons in the suprachiasmatic nucleus to AgRP neurons in the ARC via *Trh*^+^ excitatory neurons in the DMH [166]. Circadian activation of AgRP neurons may be important to align eating with optimal environmental conditions and proactively prevent energy deficit during the rest phase [166]. These results contribute to the emerging idea that homeostatic mechanisms like feeding are not only reactive, but our future needs are also anticipated, and corrective measures are taken proactively [166–173].

Whole-brain imaging of transparent tissues is not only useful for characterizing cell types as discussed above, but can also be used to map and quantify brain activation patterns in response to stimuli at single-cell resolution using c-Fos expression as a proxy for neuronal stimulation [74, 174–183]. While this method provides an unbiased approach to study the whole brain, several recent studies have investigated the consequences of stimuli relevant to the hypothalamus, such as weight-reducing drugs [74, 180]. A 5-HT_2C_ receptor agonist (lorcaserin), cannabinoid CB_1_ receptor agonist (rimonabant), dopamine D_2_ receptor agonist (bromocriptine), dual noradrenaline-serotonin reuptake inhibitor (sibutramine), GLP-1 receptor agonist (semaglutide), MC4R agonist (setmelanotide), and trace amine-associated receptor 1 agonists (ulotaront and RO5263397) have been administered to mice to identify their neuronal activation signatures [74, 180]. Unsurprisingly, these drugs caused differential activation of brain areas that regulate energy homeostasis and hedonic eating, including the PVN, DMH, ARC, and LHA of the hypothalamus and other regions in the brainstem, amygdala, thalamus, and cortex [74, 180]. These studies are crucial to understand the drug mechanisms underlying decreased body weight and can inform future drug design to provide more effective and sustained weight loss in patients with obesity.

#### 4.2.4 Neuropeptide function

Neuropeptides are released by hypothalamic neurons to regulate food intake and energy expenditure in response to hormonal, metabolic, and external signals. Neuropeptides function by binding to G-protein coupled receptors and increasing or decreasing intracellular cyclic adenosine monophosphate (cAMP) [184–187]. It was previously unknown how hunger and satiety neuropeptides regulated cAMP and influenced the function of melanocortin-4 receptor (MC4R) neurons in the PVN because it was not feasible to directly measure cAMP levels in neurons *in vivo* [184]. However, new tools were developed to induce and track endogenous neuropeptide release from AgRP and POMC neurons and manipulate cAMP levels in individual neurons in mice [184]. They found that release of NPY and alpha-melanocyte stimulating hormone (α-MSH) was all-or-none, stochastic, and affected multiple neurons within approximately 100 µm [184]. NPY released by AgRP neurons decreased cAMP and α-MSH released by POMC neurons increased cAMP in MC4R neurons in the PVN (PVN^MC4R^) [184]. Reducing cAMP in PVN^MC4R^ neurons caused increased food intake and obesity in mice [184]. These new techniques aided understanding the biochemical mechanisms by which neuropeptide signaling influences feeding behavior and resulting physiological changes [184].

#### 4.2.5 Neural feeding circuits

Animal models provide the opportunity to manipulate specific cell types within the complex *in vivo* environment and study their effects on behavior and physiology. Chemogenetics and optogenetics are two ways to modulate neurons with cell type- and region-specificity [188]. With chemogenetics, neurons of interest are genetically modified to express a designer receptor that can be activated by a drug, causing neuron activation or inhibition [188]. Optogenetics involves neurons of interest that are genetically modified to express a light-sensitive ion channel so the neurons can be depolarized or hyperpolarized with pulses of light [188]. Optogenetics requires chronic intracranial implants but allows for precise temporal control of neuron manipulation, while chemogenetics lacks this control but does not require implants and neuron manipulation can be sustained for several hours with a single drug administration [188]. During neuron manipulation, it is important to consider the duration, intensity, and with optogenetics, frequency, used to induce changes [188]. Chronic or acute manipulation can produce heterogeneous responses and it may be best to mimic “normal” neurophysiology of the targeted neurons [188]. Chemogenetic and optogenetic tools can be used to unravel intricate neural circuits that control feeding which are not fully understood.

Mutations in *BDNF*/*Bdnf* or its receptor, *NTRK2*/*Ntrk2*, cause extreme obesity in mice and humans [189–194]; however, the mechanism by which brain-derived neurotrophic factor (BDNF) neurons control feeding and body weight was unclear. A 2024 study reported that there is a simple circuit involving BDNF neurons in the VMH that are functionally downstream of arcuate AgRP and POMC neurons and project to premotor sites in the brainstem that regulate jaw movement, consummatory behavior, and feeding [195]. Using optogenetic activation and inhibition of BDNF neurons in the VMH (VMH^BDNF^), they were found to be a distinct population of glutamatergic neurons that suppressed both homeostatic and hedonic feeding [195]. VMH^BDNF^ neuron activity was decreased during food consumption and increased when food was in proximity but not consumed [195]. As these neurons receive inputs from arcuate AgRP and POMC neurons, they can sense the energy state of an animal and regulate consummatory behaviors accordingly [195].

Orexigenic AgRP/NPY and anorexigenic POMC neurons in the ARC of the hypothalamus have opposite effects on feeding and other metabolic responses. Downstream neural circuits responsible for integrating signals from these neurons have not yet been fully characterized [196]. Recent work investigated these circuits using chemogenetics to simultaneously activate AgRP neurons and inhibit POMC neurons or manipulate each neuron population in isolation in male and female mice [196]. They found that food intake was regulated by the additive effect of AgRP neuron activation and POMC neuron inhibition, but insulin sensitivity and gluconeogenesis were regulated by isolated activation of AgRP neurons and inhibition of POMC neurons, respectively [196]. They also identified *Npy1r*^+^ neurons in the PVN that respond to simultaneous input from AgRP and POMC neurons and then go on to activate *Th*^+^ neurons in the nucleus tractus solitarius to promote food intake [196]. Overall, this work informs our understanding of how feeding is precisely regulated by simultaneous and opposing functions of AgRP and POMC neurons.

#### 4.2.6 Novel cell types

Leptin is a hormone secreted by adipose tissue that binds to its receptor (encoded by *Lepr*) on orexigenic AgRP/NPY and anorexigenic POMC neurons in the ARC of the hypothalamus to suppress food intake. AgRP**/**NPY and POMC neurons have opposite effects on body weight but have key functional differences that mean they’re not exact counterparts [197]. One of these key differences is that AgRP/NPY neurons rapidly promote food intake but POMC neurons have a minimal effect on acute food intake [198–200], suggesting there may be a missing population of leptin-responsive neurons that rapidly suppress food intake [197]. A recent study performed single-nucleus RNA-seq on adult mouse arcuate neurons and identified a novel population of inhibitory GABAergic neurons marked by *Bnc2* and *Lepr* expression [197]. Further characterization of *Bnc2*^+^ neurons in mice determined that they are activated by leptin, respond to food-related sensory cues and food consumption, acutely suppress appetite and regulate peripheral glucose homeostasis, and inhibit AgRP/NPY neurons [197]. Another study also identified a leptin-responsive neuron population in the ARC, *Pnoc*^+^ neurons, that mediated diet-induced hyperphagia and obesity in mice [201]. Mouse models were crucial for identifying and characterizing novel hypothalamic cell types involved in feeding behavior.

Macaques are also used as a model system for scientific research and provide advantages over rodent models as their brain structure and genome are more similar to humans. A 2024 study characterized the role of GABAergic neurons in the LHA of macaques and found that their activation increased naturalistic goal-directed behaviors and food motivation, predominantly for palatable food [202]. While these neurons have been studied using rodents [203–211], this was the first study that was able to clearly demonstrate the role of the hypothalamus in goal-directed eating behavior in non-human primates using neuromodulation [202]. These findings are especially important as maladaptive feeding and highly palatable foods are major contributors to modern obesity [202].

#### 4.2.7 Novel feeding genes

Animal models can be used for forward genetic screens, where random mutations are introduced into the genome and animals that display a phenotype because of a mutation are studied further to identify previously unknown gene functions. A forward genetic screen was recently performed in mice to identify new genes and pathways associated with feeding [212]. They identified a missense variant in a G protein-coupled receptor gene, *Gpr75*, that did not affect GPR75 expression but resulted in lean mice [212]. For further validation, they generated *Gpr75* knockout mice and observed decreased food intake, resulting in decreased fat and body weight with high-fat diet feeding [212]. They also investigated the mechanism of GPR75 and determined that *Gpr75* was primarily expressed in neurons across various brain regions and GPR75 interacted with guanine nucleotide-binding protein G alpha q (Gα_q_), to activate several signaling pathways after high-fat diet feeding [212]. Wildtype GPR75 localized to primary cilia in mouse cells but the identified missense mutant form that decreased food intake failed to localize in the cilia, suggesting that localization is important for the function of GPR75 in regulating feeding [212]. Large-scale human exome sequencing has identified mutations in *GPR75* that are associated with lower BMI [138]; however, it is currently unknown if human GPR75 similarly localizes to primary cilia [212]. Future efforts should be focused on studying GPR75 in humans and identifying GPR75 ligands that could potentially regulate food intake [212].

Another recent forward genetic screen in mice identified two missense mutations in a different G protein-coupled receptor gene, *Gpr45*, that resulted in increased food intake and obesity [213]. GPR45 was highly expressed in the hypothalamus and the wildtype protein localized to primary cilia while the mutant forms remained in the cytoplasm [213], highlighting the importance of protein localization for its function, similar to GPR75 [212]. Targeted deletions of *Gpr45* indicated that GPR45 functions in PVN neurons to regulate feeding behavior [213]. GPR45 recruited guanine nucleotide-binding protein G alpha s (Gα_s_) into primary cilia, which enhanced MC4R signaling and activated ADCY3 to increase ciliary cAMP and suppress appetite [213]. Human *GPR45* shows high similarity to its mouse homolog and should be studied to determine if it could be the target of novel obesity therapeutics [213].

## 5 Conclusions

In conclusion, the maturation of several technologies that range in scale from single genes and proteins to whole genome, transcriptome, proteome, and other ‘omics’ has allowed us to understand more about the hypothalamus than ever before. The continued improvement and development of methodologies to better integrate these multiomic approaches in a spatial context will be important, as we strive to better understand function. As we look to the near horizon, in the big data ‘omics’ world, more is often more. Specifically, hypothalamic atlases from more model organisms will allow more sophisticated cross-species comparisons to uncover more about human biology. Finally, regarding humans, the hypothalamic data generated to date have come from healthy, mostly European-derived subjects. Thus, further studies will need to include different ethnicities, at different ages, not only in health, but also suffering from different diseases. After all, in order to understand human biology, we need to study all humans.

## Data Availability

No datasets were generated or analyzed during the current study.
